# Supplementary material for: Are carbon emissions trading and green financial instruments synergistic? -Comprehensive quantitative research based on content analysis
Source: PLoS One. 2024 Mar 7;19(3):e0298601. doi: 10.1371/journal.pone.0298601 (PMC10919601; doi:10.1371/journal.pone.0298601)
Supplement: S1 Data — (ZIP) [file pone.0298601.s001.zip › Data/地级市Z_F工作报告.zip!/║╙─╧/╜╣╫≈╩╨/└┤╫╘╣1⁄2╓┌║┼í╛┬φ┐╦╩2╛▌═°í┐_2017.docx]

各位代表：

现在，我代表市人民政府，向大会报告工作，请予审议，并请各位政协委员和列席人士提出意见。

一、2016年工作回顾

过去的一年，面对复杂的外部环境和繁重的发展任务，在省委、省政府和市委的坚强领导下，全市上下深入学习贯彻习近平总书记系列重要讲话精神和党中央治国理政新理念新思想新战略，统筹推进“五位一体”总体布局和协调推进“四个全面”战略布局，坚持以新发展理念为引领，主动适应经济发展新常态，突出供给侧结构性改革，牢牢把握经济工作主动权，围绕建设“四个焦作”、早日跻身全省“第一方阵”，迎难而上、砥砺前行，较好地完成了各项目标任务，实现了“十三五”良好开局。

——综合实力再上台阶。全市生产总值2082.6亿元，增长8.3%，迈上新的台阶。一般公共预算收入124.2亿元，增长7.9%（按营改增前可比口径），比年初目标高0.4个百分点。规模以上工业增加值1212亿元，增长7.9%。社会消费品零售总额698.9亿元，增长11.9%。

——转型升级稳步推进。高技术产业增加值增长17.9%，高于规模以上工业增加值增速10个百分点。服务业增加值增长10.1%，分别高于GDP、第二产业增加值增速1.8、2.4个百分点，对经济增长贡献率达到40.4%，动能转换初见成效。

——城乡建设步伐加快。成功创建国家森林城市、“宽带中国”示范城市，入选全省海绵城市建设示范试点市、国家全域旅游示范区创建单位，人居环境持续改善，常住人口城镇化率达到56.45%，比上年提高1.6个百分点。

——改革创新亮点纷呈。成功创建全国创新驱动示范市、全国首批社会保险标准化建设先行城市，获批全省内贸流通体制改革试点市。“探索困难群众大病补充保险的焦作样本”入选全国推进医改服务百姓健康十大新举措，并在全省复制推广。“334楼院协商治理模式”被评为全国社区治理十大创新成果。

——人民生活不断改善。民生支出166.5亿元，占一般公共预算支出76.6%。居民人均可支配收入增长7.1%。公共服务水平持续提升，社会大局和谐稳定。

主要经济指标增速由近年来多处于全省倒数二、三位，全部走出后五位，部分指标跃升全省前列。我们主要做了以下工作：

（一）加强战略谋划，五大发展理念持续深化。牢固树立和贯彻落实五大发展理念，着力谋大事、办急事、破难题，开展了一系列导向性、针对性、创新性的基础工作和战略谋划。

完善顶层设计。着眼破除发展中的体制机制障碍，出台支持工业发展28条意见、脱贫攻坚“1+16”政策、城市建设“1+N”政策体系、推动房地产市场健康发展15项举措、加快金融产业发展意见、“1+6”人才制度、规划大企业集团培育等一系列政策措施，着力打造全省先进制造业基地、健康养生基地，构建支撑发展的四梁八柱。

协调破解难题。针对城市建设与经济社会发展不适应、工作谋划与工作推进不匹配等问题，理顺市、区土地使用权出让收支管理体制，落实各方责任，凝聚各方力量，棚户区（城中村）改造、南水北调城区段绿化带综合改造提升、城市大外环等重大工程全面启动。发挥金融挂职干部作用，运用PPP等新模式，运作资金47亿元，为城市建设等提供了有力的资金保障。

践行绿色发展。面对地理气象条件差、输入性污染和累积性污染叠加等多重困难，树牢绿色理念，健全责任体系，以壮士断腕的决心，严肃执纪、严格执法，强力推进大气污染防治攻坚、北山治理、“污染围城”整治等，一些多年顽疾得到有效治理，践行绿色发展、利于人民健康、倒逼产业转型成为广泛共识和自觉行动。

突出开放合作。将开放合作作为焦作发展的生命线。眼睛向上，加强与国家部委、省直厅局的沟通对接，在海绵城市建设、综合交通体系建设等方面争取了许多项目，申建了一批试点城市和开放平台，争取国家资源枯竭城市转型资金3.78亿元。眼睛向内，重点围绕培育大企业集团等，帮助企业内引外联、延链补链、做大做强，为经济增长提供重要支撑。眼睛向外，成为中德工业城市联盟首批加盟城市，成功举办中原经济协作区第二十四届市长联席会议，与扬州、郑州航空港、华夏幸福基业、建行河南分行等合作，促成了一批基地型、中心型项目落地。

坚持共建共享。在财政收支矛盾突出的情况下，大幅压减“三公经费”，持续增加民生支出，突出办好省市重点民生实事，投入41亿元，实施28项重点民生工程，解决了一批群众关心的重点难点问题。

（二）突出精准施策，经济平稳向好势头持续巩固。面对经济下行压力，把稳增长作为突出任务，抓住扩投资、强二产、促三产等关键环节，精准发力、综合施策，促进经济运行稳中向好。

狠抓项目建设。把项目建设作为稳增长的重要抓手，集中开工项目333个，完成投资287亿元。坚持观摩点评，强化跟踪督导，多氟多新能源汽车动力总成、华夏幸福产业新城等281个重大项目开工建设，佰利联10万吨氯化法钛白粉、蒙牛乳业高端奶等43个重大项目竣工投产。固定资产投资完成2198亿元，增长16.9%，居全省第二位，有效投资的关键作用充分发挥。

夯实载体平台。把“三区”建设作为加快结构调整、促进转型升级的具体抓手和实现路径，四次集中观摩，强力督导推进。高新区成功创建国家科技服务业区域试点。5个产业集聚区总体规划调整方案、6个服务业“两区”产业发展规划调整方案通过评审，分别新增规划面积27.8、5.9平方公里。全市产业集聚区完成固定资产投资1677亿元，主营业务收入4189亿元；服务业“两区”43个千万元以上项目完成投资50亿元，万达商业广场主体完工。孟州产业集聚区进入全省“十强”，温县、武陟产业集聚区进入全省“十快”，“十强十快”数量全省第一，解放特色商业区进入全省“十先进”，科学发展载体建设走在全省前列。

强化运行调节。发挥好政府有形之手，实行周例会、月调度、季分析，深入开展产销、银企、用工、产学研对接，加大实体企业扶持力度。建立债权人委员会制度，新增贷款142.7亿元。应急转贷平台为83家企业提供支持资金9.6亿元，市级政策性融资担保平台为401家企业提供担保贷款119.1亿元。新增7家新三板挂牌企业，总数居全省第四位；246家企业在中原股权交易中心集体挂牌，总数跃居全省第二位。新增直接融资208.5亿元。“四上企业”入库796家。新获批建设用地9827亩。保持了经济平稳向好态势。

大力优化服务。树立“有事必服、无事不扰”理念，开展政策进千企活动，建立市级领导分包机制，实行重点企业首席服务员等制度，建立服务工业、服务业、农业等六本台账，因企施策、对症施治，帮助企业解决了一大批遗留问题和现实难题。支持多氟多动力总成项目财政性工业产业基金1亿元，2年内无偿使用。闲置5年的金叶醋酸纤维公司全面恢复生产。

（三）加快转型升级，供给侧结构性改革持续加强。制定并实施工业转型升级行动、服务业发展行动、农业供给侧结构性改革方案，积极落实“去降补”任务，主动扩大有效供给，经济整体素质明显提升。

突出大企业集团培育。启动大企业集团培育工程，实施110个工业转型升级项目，完成投资150.4亿元。推动风神轮胎与倍耐力成功整合，打造国内第一、世界领先的高端轮胎制造企业；佰利联成功收购龙蟒钛业，钛白粉规模跃居亚洲第一、世界第四。完成卓立、淼雨破产重整。削减煤炭过剩产能151万吨。帮助工业企业降低成本2.1亿元。新增省级企业技术中心1家、省级工程技术研究中心8家、高新技术企业20家。获得国家科技进步奖1项、省级科技进步奖18项。多氟多、中内配、风神轮胎成为全省首批创新龙头企业。工业转型升级迈出坚实步伐。

加快现代服务业发展。积极推进国际文化旅游名城建设，陈家沟太极拳、温县铁棍山药及其制品通过国家生态原产地产品保护评定，云台山获批省级旅游度假区，全市旅游综合收入增长13.6%。新引进金融机构5家，金融业增加值增长8.9%。电商交易额711.4亿元、增长30.5%，获批中国（郑州）跨境电子商务综合试验区试点城市。德众大罗塘国际农产品物流中心等项目加快推进，河南进口肉类指定口岸焦作查验场成功获批。消化库存商品住房216.7万平方米。服务业占GDP比重达到34.6%，比上年提高1.5个百分点，产业结构优化升级取得新进展。

提升现代农业体系。夏粮单产全省第一，获得“中国优质小麦之都”授牌，全国首家小麦博物馆建成开馆。新建高标准粮田18万亩，建成都市生态农业园区17个，农业产业化集群销售收入609.7亿元。农民合作社、家庭农场等新型经营主体加快发展，农村土地流转面积124.9万亩，占家庭承包经营耕地面积54.3%，分别高于全国、全省22、17个百分点。平安种业、云台山农业科技公司进入全国首批星创天地名单，修武成为首批国家农产品质量安全县，温县成为全国农村产业融合发展试点示范县。现代农业生产体系、经营体系和产业体系加快构建。

（四）实施城建带动，现代城镇体系建设持续推进。围绕提高中心城市首位度，谋划推进重大项目，城乡建设取得新突破。

全面启动中心城区三年改造提升行动。争取政策性银行资金，运用PPP等多元化筹资模式，与中建三局、中建七局、中铁七局、省投资集团等央企省企开展战略合作。以中原路、东海大道为切入点，简化审批流程，创新建设模式，大力推进十大基础设施建设项目，拉开了城市建设全面提质提速的序幕。

加强城市规划建设管理。全面开展城市设计，完成地下空间开发利用等一批专项规划编制。实施了一批城市道路、水电气暖、污水处理等工程，完成城建投资14.3亿元。新增绿色环保公交车326台。深入开展城市“双创”，省级卫生城市通过复审，城市管理水平不断提升。

加快综合交通体系建设。焦作至荥阳黄河大桥及连接线工程开工建设，郑云高速全线贯通。焦作直达上海高铁开通运行，太焦铁路全线开工，火车站南广场、南站房加快建设，焦济洛城际铁路等项目前期工作进展顺利，“十”字形高铁项目正在推进，高铁经济效应开始显现。

积极推进美丽乡村建设。出台实施方案，整治农村环境，完善基础设施，新改建农村公路521公里，改造农村危房1611户。孟州莫沟村被命名为中国美丽休闲乡村，博爱小底村成为全国一村一品示范村，温县赵堡镇入选第一批中国特色小镇；有20个村入选全省美丽乡村建设试点，美丽乡村竞相发展。

（五）深化改革开放，动力活力持续释放。把改革开放作为加快发展的根本之策，双轮驱动、共同发力，不断拓展新空间、释放新红利。

全面深化重点领域改革。深化国企改革，积极推进23家国有企业“三供一业”办社会职能分离移交，170多个职工家属区移交工作进展顺利。深化行政审批制度改革，启动国家级行政审批服务标准化试点市创建，深化并联审批，试行模拟审批，取消68项中介服务事项，砍掉43项无谓证明。全面推开营改增，实现整体减税2.6亿元。稳步推进事业单位分类改革，77家行业协会商会与行政机关脱钩。农村土地确权登记颁证、市县两级车改等基本完成。深入实施商事制度改革，新增市场主体3.4万户。

积极实施开放带动战略。成功举办十一届豫商大会，创下参会商会最多、参会代表最多、签约金额最多等纪录。重点赴长三角、珠三角、京津冀等地招商，引进云台山与灵山集团文化旅游合作等一批重大项目。新引进省外境内项目497个，居全省第一位。实际利用外资8.3亿美元，居全省第三位。进出口总额19.5亿美元，居全省第二位。开放招商为经济发展注入了强大活力。

（六）狠抓污染防治，环境质量持续改善。突出科学治污、精准治污、依法治污、全民治污，美丽焦作建设步伐加快。

重拳治理大气污染。作为政治任务、民生工程、发展要求，铁腕开展大气污染防治攻坚战，建立“1+6+7”制度体系，强力实施“六控”，严格应急管控，全市27台燃煤发电机组全部实现超低排放，比国家要求提前2年；1220台10蒸吨以下燃煤锅炉全部完成拆改，比省要求提前1年；取缔黑加油站764家；淘汰黄标车和老旧车7732辆，PM10、PM2.5浓度和空气质量优良天数实现“两降一升”。深入开展北山治理，非法采矿点、非法矿产品经营加工点全部拆除，完成矿山地质环境治理2.5万余亩。集中整治“污染围城”，清运平整垃圾338万立方米，关停“土小”企业190家。大气污染防治、北山治理、“污染围城”整治三场硬仗取得阶段性重大胜利。

大力推进节能减排。加快气代煤、电代煤进程，在全省率先推广清洁煤替代应用，市本级财政补贴1500万元，实现中心城区全覆盖。加强能源、水资源消耗总量和强度控制，加快园区循环化改造升级，全市单位生产总值能耗下降7%。

全面加强生态建设。建立空气质量生态补偿机制，制定生态环境损害责任追究办法。积极建设水生态文明城市，启动海绵城市规划、黑臭水体治理，推进绿色廊道建设，完成森林资源培育21.4万亩，森林覆盖率达到31.2%。

（七）倾力改善民生，民生福祉持续增进。把保障改善民生作为首要目标，加大民生投入，完善公共服务，脱贫攻坚、就业创业、社会保障、社会事业、社会治理等民生领域取得新成果。

脱贫攻坚成效显著。贯彻落实精准扶贫、精准脱贫基本方略，健全“九个一”机制，扎实开展转、扶、搬、保、救，建立38个产业扶贫基地，1.5万贫困人口实现稳定脱贫，48个贫困村退出贫困序列。残疾人产业脱贫“焦作模式”被中残联在全国推广。

就业创业扎实推进。实施十万个就业岗位推荐等专项行动，开展职业技能培训18万人次，新增创业担保贷款7.1亿元，城镇新增就业9.2万人，新增农村劳动力转移就业3.8万人。

社会保障更加有力。有序推进城乡居民医保制度整合，补助标准提高到每人每年420元。城乡居民最低生活保障标准提高到每人每月450元、280元，补差水平分别提高到不低于248元、132元。城乡居民养老保险参保率达到99.9%。退休人员基本养老金标准平均上调8.9%，高于国家要求2.4个百分点。建立困难残疾人生活补贴和重度残疾人护理补贴制度，发放补贴3718万元。棚户区改造开工521套，货币化安置390套，基本建成保障性住房2627套。在全国率先为进城务工农民建立住房公积金专户，“公积金管理六部法”被住建部作为行业标杆在全国推广。

社会事业蓬勃发展。完成第二期学前教育三年行动计划，示范区实验小学、中心幼儿园等项目建成投用，新增国家义务教育发展基本均衡县区4个，新增省级职业教育品牌示范特色院校2所。在去年普通高考中，本科三批次上线率均高于全省平均水平，10名学生被清华、北大录取，比“十二五”5年总数还多2名，取得恢复高考以来的历史性突破，带动了基础教育的整体提升。积极推进城市公立医院改革，全面启动市直公立医院药品零差率销售，市妇幼保健院综合病房楼、二医院新区项目等加快推进。开展文化惠民工程，百姓文化超市受益群众超260万人次。成功举办中国焦作国际太极拳高峰论坛、市十三届运动会，成为河南省第一批全民健身示范城市。

治理能力全面提升。严格落实安全生产责任，扎实开展隐患排查整治，安全生产形势保持稳定。持续加大普法宣传教育力度，荣获全国“六五”普法先进城市。推进社会治理创新，健全完善社会治安防控体系，强力推进“一村一警”和社区警务建设，严厉打击各种违法犯罪活动。依法做好信访稳定工作，扎实推进矛盾纠纷源头预防和积案化解，有效防范和处置非法集资，全面加强食品药品安全监管，人民群众安全感、满意度进一步提高。

（八）加强自身建设，行政效能持续提升。深入开展“两学一做”学习教育，全面开展“近学许昌、远学扬州”活动，深入推进依法行政，扎实开展懒政怠政、为官不为问责年活动。深化“放管服”改革，发展环境明显优化。自觉接受人大及其常委会法律监督、工作监督和政协民主监督，办理人大代表议案建议133件、政协委员提案260件。加强政府廉政建设，“三公经费”下降39.7%。军民融合深度发展，妇女儿童、红十字、慈善等事业健康发展，外事侨务、民族宗教、统计调查、对台、审计、档案、史志、气象、地震、人防等工作都取得了新的成绩。

各位代表！过去一年取得的成绩来之不易。这是省委、省政府和市委正确领导的结果，是市人大、市政协、焦作军分区大力支持的结果，是全市人民团结拼搏的结果。在此，我代表市人民政府，向辛勤工作在各条战线的全市人民，向人大代表、政协委员，向各民主党派、工商联、无党派人士、各人民团体，向驻焦解放军、武警官兵和公安干警，向离退休老领导、老同志，向关心支持焦作发展的社会各界人士，表示衷心的感谢！

我们也清醒地认识到，工作中还存在不少困难和问题。一是经济下行和环保治理双重压力愈发突出，既需要在保生态中稳增长，更需要在稳增长中促转型，提高发展质量效益的任务艰巨。二是结构性矛盾更加凸显，传统优势产业多是限产限排的管控对象，产能无法释放，支撑力明显下降；新产业、新业态占比较低，尚未形成有效支撑，亟须培育壮大新的动能。三是基础设施建设不完善，公共服务体系不健全，中心城市首位度不高，承载力、吸纳力不足，辐射力、带动力不强，是我市目前发展中的一个短板。四是服务意识不强、发展环境不优等问题依然存在，有的干部不敢担当、不会作为，思想观念、能力素质不适应新形势新要求。对这些问题，我们一定高度重视，认真加以解决。

二、2017年工作安排

今年是党的十九大召开的大喜之年，是实施“十三五”规划和推进供给侧结构性改革的深化之年，是深入贯彻落实省市党代会精神的开局之年和推动我市早日跻身全省“第一方阵”的关键之年，做好今年工作责任重大。当前，世界经济复苏乏力，国内经济“三期叠加”，焦作自身也面临不少困难。但我们更应看到，焦作正面临着新的重大机遇。

一是新战略赋予焦作新定位。国家出台《促进中部地区崛起“十三五”规划》《中原城市群发展规划》，中原城市群成为国家级七大城市群之一，郑州国家中心城市地位确立，赋予焦作建设郑州大都市区门户城市的新定位，为我们借势郑州、融合郑州，错位发展、对接发展提供了条件。只要我们抢抓机遇、勇于担当，就一定不辱使命，不辜负省委、省政府和全市人民的重托。

二是新经济孕育焦作新发展。当前，供需结构发生深刻变化，新技术、新业态、新模式不断涌现，为我们发展 “四种经济”，推进产业结构优化升级提供了更大空间。焦作拥有较为完整的产业体系，拥有一批优秀企业家，拥有一支高素质产业工人队伍，拥有一批大项目好项目。只要我们主动作为、乘势而上，就一定能在新一轮竞争中赢得先机。

三是新目标凝聚焦作新动力。省十次党代会提出建设经济强省、打造“三个高地”、实现“三大提升”的战略目标，市十一次党代会确立了实施“三大战略”、建设“四个焦作”、早日跻身全省“第一方阵”、在中原崛起中更加出彩的奋斗目标，全市上下思想空前统一、士气空前高涨，齐心协力抓发展的大好局面已经形成。只要我们深入贯彻落实习近平总书记打好“四张牌”要求，打好 “四大攻坚战”，就一定能够把宏伟蓝图变为美好现实。

今年政府工作的总体要求是：全面贯彻党的十八大和十八届三中、四中、五中、六中全会精神及中央经济工作会议精神，更加紧密地团结在以习近平同志为核心的党中央周围，统筹推进“五位一体”总体布局和协调推进“四个全面”战略布局，按照省十次党代会、省委经济工作会议和市十一次党代会、市委经济工作会议的安排部署，坚持以新发展理念为引领，坚持稳中求进工作总基调，坚持以提高发展质量和效益为中心，坚持深化供给侧结构性改革，坚持发挥优势打好“四张牌”，坚持创新驱动、开放带动、生态立市三大战略，突出以“一赛一节”为工作主线，着力做到稳增长、促改革、调结构、惠民生、防风险的平衡，加快建设“四个焦作”，在早日跻身全省“第一方阵”进程中迈出新步伐，以优异成绩迎接党的十九大胜利召开。

今年经济社会发展主要预期目标是：生产总值增长8.5%左右，规模以上工业增加值增长8.5%，第三产业增加值增长9.5%，一般公共预算收入增长8%左右，社会消费品零售总额增长12%，全社会固定资产投资增长16%，进出口总值保持稳定，居民收入与经济增长基本同步，城镇新增就业6万人以上，节能减排、环境保护等约束性指标完成省定任务，发展质量和效益明显提高，结构性改革取得实质性进展。

今年，全市政府系统以“质量、效率、法治”为要求，深入开展落实年活动，切实增强质量意识、效率意识和法治意识，紧盯目标、强化落实，确保完成全年各项任务。重点抓好以下工作：

（一）发挥市场决定性作用，促进经济稳定增长。用活市场无形之手，把扩大有效投资作为稳增长的主要支撑，持续释放需求潜力，促进有效投资和消费升级良性互动，推动经济发展行稳致远。

加强项目谋划建设。坚持项目为王，围绕国家宏观政策和投资走向，谋划推进一批龙头型、科技型、基地型、品牌型“四型”项目，争取更多项目纳入国家、省计划盘子。今年安排市重点项目178个，总投资1057亿元，计划年度完成投资296亿元。加强跟踪督导，早日达产达效。

强化发展载体支撑。加快示范区创新发展，推进主导产业集群培育、城市功能综合连片开发，积极构建城乡一体的基础设施体系，着力打造重要经济增长极、城市新亮点和生态宜居地。促进产业集聚区提质转型，突出抓好集群培育、功能完善、产城融合、机制创新，实现全市产业集聚区主营业务收入4600亿元、完成固定资产投资1900亿元。推动服务业“两区”提速增效，创新开发建设模式，实现新开工千万元以上项目20个以上、完成固定资产投资100亿元。积极争强夺快晋星，争取更多产业集聚区、服务业“两区”进入全省“十强十快十先进”。

努力扩大民间投资。以国家支持鼓励民间投资为契机，引导民间资本进入棚户区（城中村）改造、市政公用事业、城市基础设施、社会服务等领域，促进民间投资回升。落实《焦作市促进民间投资健康发展实施方案》，在产业发展、融资支持、土地供应等方面主动服务，优化民营经济发展的政策、政务和法治环境，推动民营企业做大做强，促进民营经济蓬勃发展。

推动消费加快升级。实施扩大和促进消费专项行动，持续扩大传统领域消费，加快发展文化休闲、体育健身等新型消费业态。扎实推进全省内贸流通体制改革试点市建设，编制城区专业市场等专项规划，推进农产品批发市场、农贸市场升级改造，完善城乡市场体系，释放居民消费潜力。

大力发展实体经济。认真落实降成本政策措施，加大减税、降费、降要素成本力度。创新社会化融资机制和服务模式、服务产品，加快打造“政投保贷介”五位一体的公共服务平台，有效缓解中小微企业融资难、融资贵问题。引导企业加强财务管控，优化债务结构，降低企业杠杆。鼓励支持企业引进来战略重组，支持有条件的企业走出去并购重组，推动本土企业间战略合作，实现强强联合、裂变扩张。

（二）加快转型升级，提升供给质量效率。打响发展转型攻坚战、持久战，突出以推进供给侧结构性改革为经济社会发展的主线，不断优化产业结构，扩大有效和中高端供给，加快构建具有核心竞争力的现代产业体系。

加快工业转型升级。坚持工业强市不动摇，全力打造全省先进制造业基地。加快发展先进制造业。深入实施“443”产业提升行动，提质强大装备制造、汽车及零部件、食品、现代化工等优势产业，打造4个千亿级产业集群；培育壮大生物医药、电子信息、新材料、新能源等新兴产业，努力打造经济增长新的支撑点；以改工艺、改装备、改产品为重点，改造升级铝、能源、轻工等传统产业，着力调高技术水平、调强制造能力、调优产业结构、调长产业链条，推动产业产品向价值链中高端跃升。积极培育大企业集团。实施“1020工程”，加快培育10家超100亿元、20家超50亿元的大企业集团。出台加快培育大企业集团实施意见，着力抓龙头、强链条、建集群，扶持骨干企业做大做强，尽快打造一批主业优势强、发展态势好、核心竞争力和带动力大的企业集团，争取今年新增1~2家100亿企业、2~3家50亿企业。抓好十大工业项目建设。争取多氟多新能源汽车动力总成、蒙牛乳业百亿产业集群部分竣工投产，超威新型蓄电池项目一期竣工投产。加快推进中州铝业转型示范园、隆丰皮草皮毛综合加工产业园建设，开工建设佰利联20万吨氯化法钛白粉扩建项目、焦作万方高端铝深加工和中原内配发动机关键零部件项目。力争风神股份全球工业轮胎研发中心及倍耐力高性能轿车子午胎产品升级等项目开工建设。着力提升产业素质。推进数字化车间和智能工厂自动化改造，提升制造业数字化、网络化、智能化水平。加强企业家培训，开展质量提升行动，大力弘扬“工匠精神”，加强品牌建设，增强产品竞争力。有序化解过剩产能。今年再退出煤炭产能66万吨。用市场、法治的办法，稳妥处置“僵尸企业”，倒逼环保停工停产企业转型转产，不让落后产能挤占先进产能。

大力发展现代服务业。全力创造新供给、发展新业态、培育新产业，推动服务业发展提速、比重提高、水平提升。加快文化旅游业转型发展。依托“山水焦作、太极圣地”两大品牌，围绕创建国家全域旅游示范市，实施八大旅游提升工程，推动国际文化旅游名城建设，加快二次创业步伐。重点抓好旅游产品开发、要素完善、宣传营销、服务监管等工作，加快云台山综合旅游度假区、中国太极镇、老家莫沟二期、月山寺禅修养生小镇、当阳峪绞胎瓷小镇等项目建设。积极培育文化时尚健康快乐旅游经济新业态，打造“世界太极城·中原养生地”。加快金融业突破发展。深入开展“引金入焦”工程，启动市级金融产业集聚区规划，大力发展区域性金融机构，吸引各类金融机构在我市设立法人总部、地区性总部或后台业务总部，力争引进5家金融机构。支持焦作中旅银行在依规不变注册地的前提下，走出焦作、回馈焦作，打造特色鲜明的地方商业银行。完成农信社改制任务，组建焦作市农村商业银行，探索设立金融控股平台。加强与金融机构的有效合作，充分利用政策性、开发性金融，支持重点领域、重点行业发展和薄弱环节改善，鼓励商业银行加大支持实体经济力度。推动企业进入多层次资本市场，推进云台山股份报会审核，争取2家企业启动IPO程序，新三板、四板再挂牌一批企业。鼓励企业通过首次公开发行和再融资，发行企业债、公司债，引进股权投资等多种形式，扩大直接融资规模。创办焦作金融大讲堂，提升政府金融运作能力。防范化解金融风险，确保金融市场稳定。加快电子商务创新发展。深化与阿里巴巴、苏宁、腾讯等知名电商合作，扶持壮大怀庆府、博爱人家等本土电商平台。推动跨境电商试点建设，打造德众全球购跨境进口电商平台和通港物流园跨境出口电商平台，加快建设焦作跨境电商生态圈，构建“一区多园、一园多点、多主体运行、多模式发展”的跨境电商格局。加快商贸物流业融合发展。推进焦西物流金融示范园、腾云电商产业园集中仓储和物流配送中心、鸿运智能物流园等项目，推动物流业与农业、制造业、商贸业融合联动。鼓励传统百货、连锁超市、专业市场等商贸流通企业开展网络批零业务，引导生产企业开展网络直销、网上订货，提高产供销协同运作能力。促进房地产业健康发展。结合棚户区（城中村）等改造提升工程，完善住房消费政策，努力满足群众多层次住房需求。加快建立购租并举的住房制度，推进货币化安置，做好公租房回购和分配，培育房屋租赁市场。加强房地产市场监管，改进规范物业管理，让群众买得放心、住得舒心。

促进农业提质增效。深入推进农业供给侧结构性改革，培育农业农村发展新动能，提高农业综合效益和竞争力。调整优化种养结构。推进高标准粮田建设，完善农业基础设施，提升农业机械化水平，强化气象为农服务，确保粮食总产稳定在40亿斤以上。加快发展高效种养业，按照“四优四化”要求，重点发展优质小麦、优质花生、优质草畜、优质林果。促进“四大怀药”规模化种植、标准化生产、品牌化销售，拉长产业链条、提升产业品质。加快沿黄滩区奶牛、肉牛饲养基地建设，推进布局区域化、经营规模化、生产标准化、发展产业化。提升农业产业化水平。围绕农业六大产业，开展农民创业创新行动，建成100个创效型产业村，发展1000家创新型企业，培育1万名创业型农民。加快农业观光园、生态园、创业园、示范园等园区建设，确保农业产业化集群销售收入达到650亿元。实施农产品品牌战略。大力推动农业标准化生产，加强农畜产品质量安全全过程监管，加快建立农畜产品质量安全追溯信息平台，打造一批竞争力强、影响面广的知名品牌，新增“三品一标”农产品30个，新培育标准化生产示范基地8个。

突出发展“四种经济”。紧跟产业变革方向，培育发展新业态、新经济，积蓄发展新动能。网络经济方面，深入实施“互联网+”行动，大力发展智能制造、大数据、云计算、物联网，推动蛮蛮云计算数据中心、焦作浪潮大数据创新应用中心等项目建设，推进温县千亩铁棍山药物联网应用示范工程、万亩现代农业创新园区物联网中心等项目建设，加快制造业智能化服务化、种养业智慧化精准化、服务业品质化高端化步伐，为传统产业发展插上“网络翅膀”。枢纽经济方面，加快建设焦作西站、武陟公铁物流港等项目，谋划推进博爱高铁新城等项目，大力引进供应链金融、电商物流等新业态项目，积极培育适宜在交通枢纽周边发展的现代服务业和先进制造业。创意经济方面，积极推进修武光子创客基地、百年电厂文化创意产业园等项目，大力发展与产品研发、文化旅游、咨询策划等相关的创意产业，培育打造一批创意园区、骨干企业和特色品牌。生态经济方面，加快建设国隆实业新型建筑材料等项目，大力培育节能环保、绿色建材、资源综合利用等新型生态产业。

（三）着力扩大开放，实现一招多效。强力实施开放带动战略，借船出海、借梯登高，持续提升双向开放水平。

全力抓好“一赛一节”。将“一赛一节”作为部署推进全年工作的主线，坚持节会搭台、文旅为媒、经贸唱戏，实施产业招商行动计划，组建招商团队，绘制招商图谱，运用集群招商、以商招商、驻地招商等方式，创新采用股权招商、并购招商等模式，重点针对长三角、珠三角、京津冀等先进地区先进产业，集中引进一批大项目好项目，实现招商引资新突破。扎实开展招商引资项目落地年活动，切实提高项目履约率、资金到位率、项目开工率。

加快郑焦融合发展。把郑焦融合作为开放战略的重中之重，组建专门班子，深入研究谋划，建立郑焦两市沟通协作机制，积极推进交通融合、产业融合、生态融合。对接郑州大都市区重点产业带规划布局，谋划建设郑焦产业带，研究规划南部产业新城，努力打造具有较强辐射作用和综合服务功能的大都市区重要门户。

全面深化对外开放。加快外向型经济园区建设，积极融入河南自贸区建设。加强与中德工业城市联盟会员城市交流合作，把德国产业发展的“质”嫁接到我们发展的“量”上。深化与郑州航空港战略合作，大力引进电子信息、生物医药等出口型临空产业项目。加快建设河南进口肉类指定口岸焦作查验场、沁阳鹏海保税博览城，积极申建焦作德众综合保税区。深化关检合作“三个一”模式，推动通关一体化。支持企业积极开拓“一带一路”沿线新兴市场，让焦作与世界精彩互动。

（四）实施创新驱动战略，推进发展动能转换。坚持把创新作为引领发展的第一动力，健全创新体系，建设创新焦作。

发展创新平台。以国家级高新区为载体，编制实施“一区四园”发展规划，争取尽快纳入郑洛新国家自主创新示范区拓展区。健全提升科技大市场、科技银行、装备制造技术研究中心等服务平台功能，新建一批工程技术研究中心、企业技术中心、院士工作站、重点实验室、众创空间等创新研发平台，提升大中型企业省级以上研发机构覆盖率。

壮大创新主体。落实财税、金融等支持政策，引导鼓励企业发挥主体作用，加大研发投入。实施20项省级以上科技项目，重点支持一批市场前景好、研发能力强的创新型企业，培育一批高新技术企业、科技小巨人企业和科技型中小微企业，壮大创新集群，增强核心竞争力。

完善创新机制。加快设立科技企业投资引导基金、科技创新奖励基金，建立科技经费拨改投、拨改奖机制，为万众创新提供制度保障。积极与中科院、大连理工等科研院所、高等院校、龙头企业开展科技合作，发挥河南理工大学科研优势，集聚创新资源，加强自主创新、开放创新和协同创新，促进产学研用有机衔接、一体发展。

引进创新人才。树立“不求所有、只求所用”人才观，全面落实“1+6”人才制度，确保引进高层次紧缺人才1000人以上。实施创新人才培育工程，大力培养领军型、创新型、实用型人才队伍，为全面建成小康社会提供坚强人才保证。

（五）深化重点领域改革，增强发展内生动力。坚持问题导向，着力破解发展瓶颈制约，激发市场活力和社会创造力。

深化“放管服”改革。继续推进行政审批制度改革，积极创建国家级行政审批服务标准化试点市。加快商事制度改革，实现“双随机、一公开”监管全覆盖。大力推行“互联网+政务服务”，实行“一号申请、一窗受理、一网通办”，推动行政审批和公共服务事项从实体大厅向网上办事大厅延伸。全面推行公共资源电子交易，实现网上全公开、网下无交易。

深化财税体制改革。进一步理顺市与县（市）区财税体制，优化利益共享机制，调动各级积极性。深化国地税征管体制改革，加强国地税合作。加快财税智慧云平台建设，推进社会综合治税。

深化国资国企改革。完善企业产权制度，健全法人治理结构，推动国企负责人选拔制度和薪酬制度改革，启动市场化选聘职业经理人试点，加快推进国企“三供一业”分离移交工作。健全国有资产监管方式，积极开展国有资本投资试点。

深化农村改革。落实承包土地所有权、承包权、经营权分置办法，全面完成农村土地承包经营权确权登记颁证。改革财政支农投入使用机制，加快农村金融创新。持续深化水利、供销社等改革，全面完成国有林场改革任务。

深化投融资体制改革。加快政府投融资平台市场化转型，研究设立创业投资、产业投资和新型城镇化建设基金，大力推广运用PPP模式，撬动更多社会资本和金融资本。

同时，加快电力体制改革，健全经济统计制度，推进环保执法、文化体制、盐业等改革，真正使改革成为推动发展的重要动力、破解难题的制胜法宝、惠及民生的最大红利。

（六）统筹城乡协调发展，加快新型城镇化建设。实施“四城联创”，探索符合焦作实际的新型城镇化路子，加快形成城乡协调发展新格局。

突出抓好十大基础设施建设项目。一是全面启动南水北调城区段绿化带及综合改造提升工程，加快推进征迁安置、绿化带建设等工作。二是强力推进黑臭水体治理，年底前基本消除城市黑臭水体。三是开工建设中原路项目，年底前力争开工焦温快速通道（西环路）。四是开工建设东海大道项目。五是协调推进焦作至荥阳黄河大桥及连接线等在建项目，争取南太行绕城高速、孟州至偃师黄河大桥等项目早日开工建设。六是开工建设苏蔺水厂，年底前建成主体工程，力争府城水厂早日开工建设。七是加快太焦铁路、焦作站南广场和南站房建设，谋划推进南太行旅游观光铁路项目，强力推进焦济洛城际铁路前期工作。八是完成大沙河生态治理主体工程。九是推进新河商务区尽快进入实质性建设。十是加大棚户区（城中村）改造力度。

着力提升中心城市承载能力。深入推进中心城区三年改造提升行动，加强城市规划建设管理。完成北部山区等重点区域规划设计。统筹开展城区道路新建改建、燃气、供电、供热、供水管网、地下综合管廊等建设工作，积极推进世行贷款绿色交通项目。加快海绵城市示范区建设，建成2个海绵型公园、10个海绵型游园。加快马村、中站污水管网建设，抓好雨污分流管网、防洪排涝设施建设和污水处理厂运行。加强城市地下空间开发利用，提升城市应急避险能力。完成城市执法体制改革，加强市容秩序管理、环境卫生整治、市政设施改造，推动城市管理走向城市治理。

大力实施县城建设提质工程。推动县城建设提质、提速、提效，沁阳、孟州、武陟要加大步伐，走在全省前列。上半年所有县（市）完成城乡总体规划修订编制，同步完成总体城市设计，年底前完成市政公用设施和公共服务设施专项规划编制。各县（市）要统筹地上与地下建设，规划推进供热、供气等重点城建项目，加快实现城市化提升。

加快特色小镇建设。规划建设一批具有产业、文化、旅游、社区四大城市功能的电商小镇、文化旅游小镇、健康养生小镇、商业休闲小镇，力争每个县（市）新培育1~2个特色小镇。

积极推进美丽乡村建设。坚持示范引领、梯次推进，推广“老家莫沟”经验，开展“四美乡村”建设，确保全市30%的行政村达到宜居乡村标准、10%的行政村达到美好乡村标准，让群众生活更加舒适幸福。

（七）强力实施环境治理，筑牢生态立市根基。牢固树立绿水青山就是金山银山的理念，坚持生态立市，加快建设美丽焦作。

打好环境污染防治攻坚战。进一步增强政治意识、大局意识，以更大决心、更强力度、更硬措施，统筹开展好大气、水、土壤污染防治攻坚。坚决打赢大气污染防治攻坚战。突出重点行业、重点领域、重点地区，实施污染治理提升工程，确保实现PM10、PM2.5浓度稳定下降，空气优良天数持续上升。坚决打好水污染防治攻坚战。严格落实“河长制”，系统推进水污染防治、水资源管理和水生态保护，确保4个省控责任目标断面水质达标，保障城市集中式饮用水水源和南水北调中线水质安全。扎实做好土壤污染防治攻坚战前期工作。启动土壤污染状况详查，加强污染源监管，开展好土壤环境治理修复试点工作。持续抓好北山治理和“污染围城”整治。严格执行《焦作市北山生态环境保护条例》，全面推进北山生态恢复，构筑绿色生态屏障。巩固“污染围城”整治成果，做好场地清理、绿化美化、开发建设等工作。

推进节能减排。做好碳排放权交易市场启动工作。继续开展“四个一批”专项整治，大力治理火电、钢铁、水泥、碳素、陶瓷等重污染行业企业。开展清洁生产，推广清洁能源，加快“气化焦作”建设，全面完成清洁煤替代工作。出台装配式建筑产业发展支持政策，推进商砼产业园、装配式建筑产业园规划建设，引导建筑行业与市场转型升级。积极推行垃圾分类制度，规划建设静脉产业园，推动垃圾减量化、资源化、无害化处理。

加强生态体系建设。实施国家森林城市生态提升行动，加快省级国家储备林基地试点市建设，完成森林资源培育21万亩。启动4个高速出入口提升改造、人民西路绿化和城东公园建设，完成绿色廊道建设。高标准编制全域水系规划，谋划实施一批水生态项目，做活水经济、做大水文章。

（八）切实保障改善民生，确保社会和谐稳定。坚持以人民为中心的发展思想，持续加大投入，办好民生实事，提供更公平更优质的公共服务，让全体人民共享发展成果。

让贫困群众早日脱贫。把“精准”贯穿脱贫攻坚全过程，因人因地施策，打好脱贫攻坚组合拳，推进农村低保制度与扶贫政策有效衔接，完成2.37万贫困人口稳定脱贫。

让群众享有更充分的就业。实施就业服务提升行动，重点做好高校毕业生、去产能企业分流职工、农村富余劳动力三大群体就业工作；加快创业型城市建设，推进全民技能振兴工程，大力扶持高校毕业生、返乡农民工等群体创新创业。新增农村劳动力转移就业2万人。

让群众享有更可靠的社会保障。实施全民参保登记计划，调整企业和机关事业单位退休人员基本养老金，全面完成机关事业单位养老保险制度改革。完善城乡居民大病保险制度，推进医疗费用异地就医即时结算，实现城镇居民医保与新农合制度的完全融合，财政补助标准提高到每人每年450元。落实好特困人员供养制度，深入推进困难群众大病补充医疗保险制度，将贫困群众全部纳入保障范围。城乡低保月人均财政补差水平分别提高到不低于250元、142元。引导社会资本进入养老服务业，健全服务体系，提升服务质量。

让群众享有更完善的公共服务。推进学前教育普惠发展，巩固提高义务教育均衡发展水平，持续提升高中教育质量，加快推进映湖路小学、示范性综合实践基地、韩公教育园区中学等项目建设。大力发展职业教育和民办教育，支持河南理工大学争创一流学科，焦作大学、焦作师专等提升办学水平，努力让学子们享有更优质的教育。大力推进市域综合医改试点市建设，统筹推进医疗、医保、医药联动改革，全面推进城市公立医院改革，加快推进市级医院建设项目，加强重大疾病防控和儿童、妇女、老年人、残疾人等重点人群健康服务。加快基层综合性文化服务中心建设，继续实施好百姓文化超市、舞台艺术送基层等惠民工程。努力做好太极拳申遗相关工作。推进新档案馆前期工作。广泛开展全民健身运动，提高体育场馆开放水平，建成一批农民体育健身工程，积极申创全国武术之乡、省级体育产业基地。

让群众享有更和谐的社会环境。深化平安焦作建设，做好做深做细社会治理工作。以“七五”普法为载体，加强法治宣传，树牢法治理念，加快法治焦作建设。积极推进社区治理和服务创新，力争3年时间在全市推开“334”楼院协商治理模式。进一步深化和巩固社会治安防控体系，持续深化打击违法犯罪行动。强化四级平台作用，深入推进“三调对接、四级联动”矛盾纠纷多元化解机制，畅通民意表达渠道，深入推进阳光信访、责任信访、法治信访。加强网络安全管理，落实安全生产、食品药品安全责任制，健全公共应急体系，依法防范和处置非法集资。让焦作更加安定团结、更加温馨和谐、更加幸福美好。

积极支持驻焦部队建设，深入开展双拥共建，推进军民融合深度发展，争创省级双拥模范城“七连冠”。促进民族团结、宗教和睦。支持工会、共青团、妇联等群团组织工作。加强外侨、对台、统计调查、审计、地震、史志、援疆等工作。

三、全面加强政府自身建设

做好政府工作，必须切实增强“四个意识”，始终在思想上政治上行动上同以习近平同志为核心的党中央保持高度一致，思想上深刻认同核心，政治上坚决维护核心，组织上自觉服从核心，行动上坚定紧跟核心，把讲政治、敢担当、重操守贯穿于政府工作全过程和各方面，确保党中央的各项决策部署不折不扣地在焦作落地生根、开花结果。在具体工作中，全市政府系统要围绕建设“四个焦作”、早日跻身全省“第一方阵”的目标，深入开展以“质量、效率、法治”为要求的落实年活动，转变政府职能，改进工作作风，撸起袖子加油干，再大困难也不变，做到为人民服务、对人民负责、受人民监督、让人民满意。

（一）践行新的理念。主动适应、把握和引领经济发展新常态，牢固树立和贯彻落实五大发展理念，坚持以提高发展质量和效益为中心，自觉做到崇尚创新、注重协调、倡导绿色、厚植开放、推进共享。紧密结合焦作实际，突出供给侧结构性改革，把贯彻新发展理念体现到政策制定、工作部署、任务落实等各方面各环节，用新理念引领新发展、推动新实践，实现焦作在新起点上有更高质量、更好效益、更加公平、更可持续的发展。

（二）提升行政效能。坚持一流标准，对确定的发展目标和重点任务，制订实施方案，建立工作台账，做到目标明确、节点清楚、标准具体、责任到位，达到可量化、可操作、可考核、可兑现，确保各项目标任务圆满完成。优化发展环境，开展企业家日活动，严格执行首问负责制、服务承诺制和限时办结制，坚决做到“有事必服、无事不扰”，更好地服务基层、服务群众、服务企业、服务项目，创造焦作质量，打造焦作效率。

（三）严格依法行政。认真贯彻全面依法治国基本方略，主动落实党政主要负责人履行推进法治建设第一责任人职责规定，依法决策、依法行政、依法治理，运用法治思维和法治方式深化改革、推动发展、化解矛盾、维护稳定，把政府活动全面纳入法治轨道。严格执行人大及其常委会决议决定，自觉接受人大法律监督和工作监督，自觉接受政协民主监督，认真办理人大议案、建议和政协提案，广泛听取民主党派、工商联、无党派人士、人民团体意见，主动接受舆论监督和社会监督。

（四）砥砺严实作风。自觉践行“三严三实”要求，始终把纪律和规矩挺在前面，认真贯彻中央八项规定精神，严格执行《准则》《条例》，严格落实党风廉政建设主体责任和“一岗双责”，持续开展懒政怠政、为官不为专项治理，驰而不息纠正“四风”，坚决整治侵害群众利益的不正之风和腐败问题，确保人民赋予的权力为人民服务。强化责任担当，改进绩效考核，完善督查制度，健全激励机制，落实“三个区分开来”，激发政府系统的使命感、责任感、紧迫感，以踏石留印、抓铁有痕的劲头抓落实，推动经济社会持续健康发展，奋力谱写决胜全面小康的新篇章。

各位代表！新使命开启新征程，新目标呼唤新作为。让我们更加紧密地团结在以习近平同志为核心的党中央周围，在省委、省政府和市委的坚强领导下，同心同德、锐意进取，加快建设“四个焦作”，早日跻身全省“第一方阵”，让焦作在中原崛起中更加出彩，以优异成绩迎接党的十九大胜利召开！
